# A Case of Secondary Trigeminal Neuropathy Due to Local Malignant Invasion of the Maxillary and Mandibular Nerves at the Skull Base: A Case Report With Review of Differential Diagnosis

**DOI:** 10.7759/cureus.24391

**Published:** 2022-04-22

**Authors:** Monica Pasala, Gyusik Park, Hassan N Kesserwani

**Affiliations:** 1 School of Medicine, University of Alabama at Birmingham, Birmingham, USA; 2 Neurology, Flowers Medical Group, Dothan, USA

**Keywords:** meckel’s cave, basal cell carcinoma, perineural invasion, perineural spread, trigeminal neuropathy, enhanced mandibular nerve, gasserian ganglion

## Abstract

Trigeminal neuropathies (TNp) are a group of well-characterized disorders that involve damage to or infiltration of the trigeminal nerve. The underlying etiology of trigeminal neuropathy can be traumatic, inflammatory, autoimmune, paraneoplastic, malignant, and very rarely infectious. We present a case of trigeminal neuropathy due to local malignant invasion of the mandibular nerve with mandibular nerve enhancement at the foramen ovale and foramen rotundum. In the process, we review various etiologies of trigeminal neuropathy associated with trigeminal nerve involvement at the foramina. We emphasize the importance of a comprehensive evaluation in patients with trigeminal neuropathy, which includes searching for perineural spread or invasion by a local head and neck malignancy, as well as ruling out an inflammatory or autoimmune etiology. Our case also demonstrates that a higher field strength magnet can reveal pathology unseen with a lower field strength magnet.

## Introduction

The trigeminal nerve, cranial nerve V, is the largest of the 12 cranial nerves and has mixed sensory and motor functions. Its origin is in the brainstem and includes three sensory nuclei (mesencephalic, principal sensory, and spinal nucleus of the trigeminal nerve) and one motor nucleus (motor nucleus of the trigeminal nerve). Sensory information travels via afferent neurons from the face to the trigeminal ganglion and distributes via the various sensory trigeminal nuclei. Sensory information, such as pain and temperature, is then relayed to the contralateral thalamus and eventually synapses in the postcentral gyrus. Motor information travels via efferent neurons from the motor nucleus directly to its targets: masseters, temporalis, and pterygoid muscles [1,2].

The trigeminal nerve divides into three branches at the Gasserian ganglion in Meckel’s cave [3]. The three branches consist of the ophthalmic nerve (V1), the maxillary nerve (V2), and the mandibular nerve (V3). The ophthalmic nerve (V1) travels through the cavernous sinus, exits the base of the skull, and enters the orbit via the superior orbital fissure. The maxillary nerve (V2) accompanies the ophthalmic nerve in the cavernous sinus as it makes its way to the pterygopalatine fossa by exiting the skull base via the foramen rotundum. At the pterygopalatine fossa, the maxillary nerve splits into many branches. A trigeminal nerve deficit (V1 and V2) and a sixth nerve palsy localize a lesion to the cavernous sinus. The mandibular nerve (V3) does not traverse the cavernous sinus but runs along and eventually exits the base of the skull via the foramen ovale into the masticator space [4].

The ophthalmic and maxillary nerves are sensory branches of the trigeminal nerve. The ophthalmic division branches into the frontal, lacrimal, nasociliary, tentorial, and dural nerves. These nerves provide sensory innervation to the cutaneous surfaces of the upper eyelids, forehead, and sides of the nose. The nasociliary nerve specifically innervates the tip and the sides of the nose, and its involvement in herpes zoster ophthalmicus is known as Hutchinson’s sign. The ophthalmic branches also innervate the mucosa of the frontal sinus, lacrimal glands, cornea, conjunctiva, ciliary body, and dura mater [5]. Damage to some of these branches, more specifically to the parasympathetic efferent nerves of the lacrimal glands, can manifest as dry eyes in Sjogren’s syndrome. The maxillary division of the trigeminal nerve branches into the infraorbital, zygomatic, greater palatine, lesser palatine, posterior superior alveolar, and meningeal nerves. These nerves provide sensory innervation to the region below the orbit and above the mouth, the maxillary teeth, and the maxillary sinus.

The mandibular division of the trigeminal nerve is unique in that it branches into both sensory and motor nerves. Sensory nerves include meningeal, lingual, auriculotemporal, inferior alveolar, buccal, and mental nerves. A mental neuropathy with numbness of the chin is a sinister sign of local malignant invasion. These nerves provide sensory information to the region below the mouth, the mandibular teeth, and the anterior two-thirds of the tongue. The motor nerves include the masseteric, deep temporal, medial pterygoid, lateral pterygoid, and mylohyoid branches. A motor nerve root supplies the tensor veli palatini, a lesion of which causes palatal myoclonus. Another motor twig goes to the tensor tympani, and injury here leads to hyperacusis [2,5]. It should also be emphasized that the mandibular branches, with the ophthalmic branches, supply the dura mater of the anterior and middle cranial fossa.

Neoplastic, autoimmune, paraneoplastic, and autoimmune processes can damage the trigeminal nerve, leading to trigeminal neuropathy (TNp) [6,7]. The characteristic findings of TNp include facial numbness and weakness of the muscles of mastication. TNp is different from trigeminal neuralgia (TN), which is characterized by episodes of sudden, very brief, severe, sharp, shooting facial pain. The initial presentation of TNp can include pain; however, with disease progression, facial numbness and masticatory muscle weakness may predominate [8]. As TNp may be the initial presentation, malignancies of the head and neck should always be in the differential diagnosis, and a high-resolution magnetic resonance imaging (MRI) of the brain and skull base with and without gadolinium enhancement is paramount [9].

## Case presentation

We present the case of a relatively healthy 80-year-old female who presented to the clinic with a several month history of recurrent sharp, shooting left midfacial pain. The pain was associated with tingling in the left half of the face and loss of taste. With initial symptom presentation, the patient went to the emergency department and was diagnosed with Bell’s palsy and was given gabapentin for the pain. At her follow-up at the neurology clinic, she presented with unimproved symptoms in addition to newly developed left lower facial weakness and occasional chewing difficulty. She denied hyperacusis. Twenty years ago, she had presented with a left midface nodule, which was excised and diagnosed as a basal cell carcinoma. She has not had a recurrence since.

Her past medical history is significant for hypertension, for which she takes lisinopril 10 mg once daily. The patient denied any significant family history. She has never smoked, does not consume alcohol, and denies using any illicit or recreational drugs. Constitutional symptoms including weight loss, fever, and malaise were not reported.

Vital signs revealed a blood pressure (BP) of 170/91 mmHg with a pulse of 81 beats per minute and oxygen saturation of 97%. The patient’s height was 5 feet and 8 inches with a weight of 185 pounds and a body mass index of 28.1 kg/m^2^. Physical examination showed a well-nourished individual who was alert, relaxed, and cooperative. The patient was oriented to person, place, situation, and time. Gait was steady with a normal base, arm swing, and turning. Heel- and toe-walking was normal with an absent Romberg sign. Speech was of normal tone, volume, and prosody.

Cranial nerve examination revealed normal extraocular motion with symmetric pupils and preserved accommodation, and visual fields were full to confrontation. There was a striking decrease in sensation to touch and pinprick over the left V2 and V3 distribution. The corneal reflex was brisk bilaterally. No masseter or temporal muscle atrophy was noted bilaterally. Left lower facial weakness was noted with depressed nasolabial fold, inability to blow the left cheek, and conspicuous sagging of the left lower face. Hearing was intact to finger rub bilaterally. The gag reflex was preserved bilaterally with a tongue that protruded to the midline. The trapezii and sternocleidomastoids were well developed and symmetric, and she executed shoulder shrug and head-turning with adequate power.

**Figure 1:**
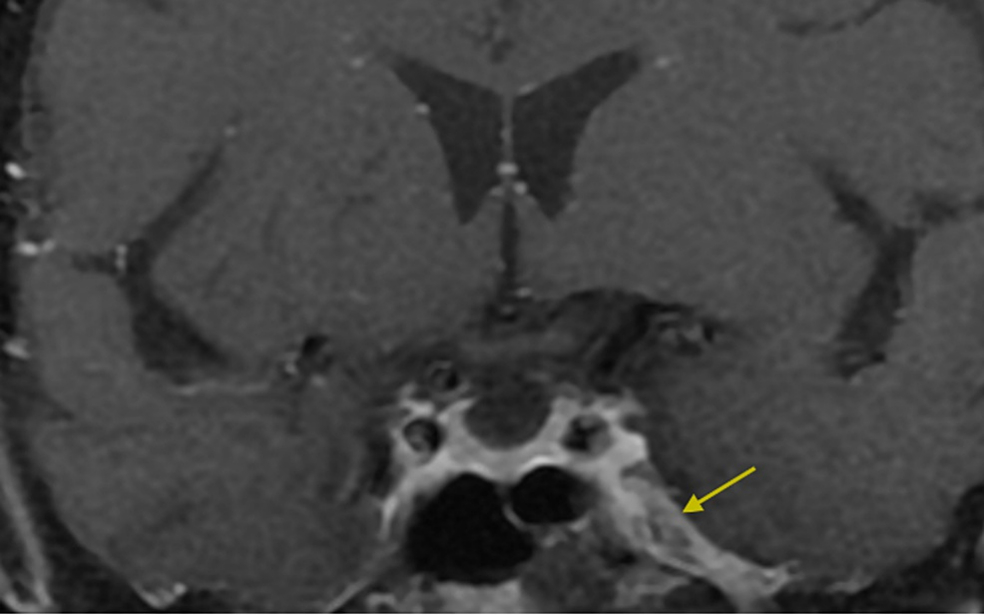
Gadolinium-enhanced coronal T1-weighted 1.5-Tesla MRI images of the brain showing mandibular nerve enhancement in the left foramen ovale (yellow arrow).

Motor examination showed good muscle bulk and tone. No pronator drift or spastic catch of the arms was noted, and the legs were of normal tone. Strength was graded at 5/5 with the Medical Research Council (MRC) rating scale in the upper and lower extremities bilaterally. Sensation to vibration, position, light touch, and pinprick was normal in the fingers and toes. Deep tendon reflexes (biceps, triceps, brachioradialis, patellar, and Achilles) were lively and bilaterally symmetric. Babinski sign was absent bilaterally. Finger-to-nose and heel-to-shin motions were normal bilaterally. An initial 1.5-Tesla MRI of the brain with and without contrast revealed an asymmetrical enhancement of the left mandibular branch of the trigeminal nerve as it extended through the foramen ovale (Figure 1).

**Figure 2:**
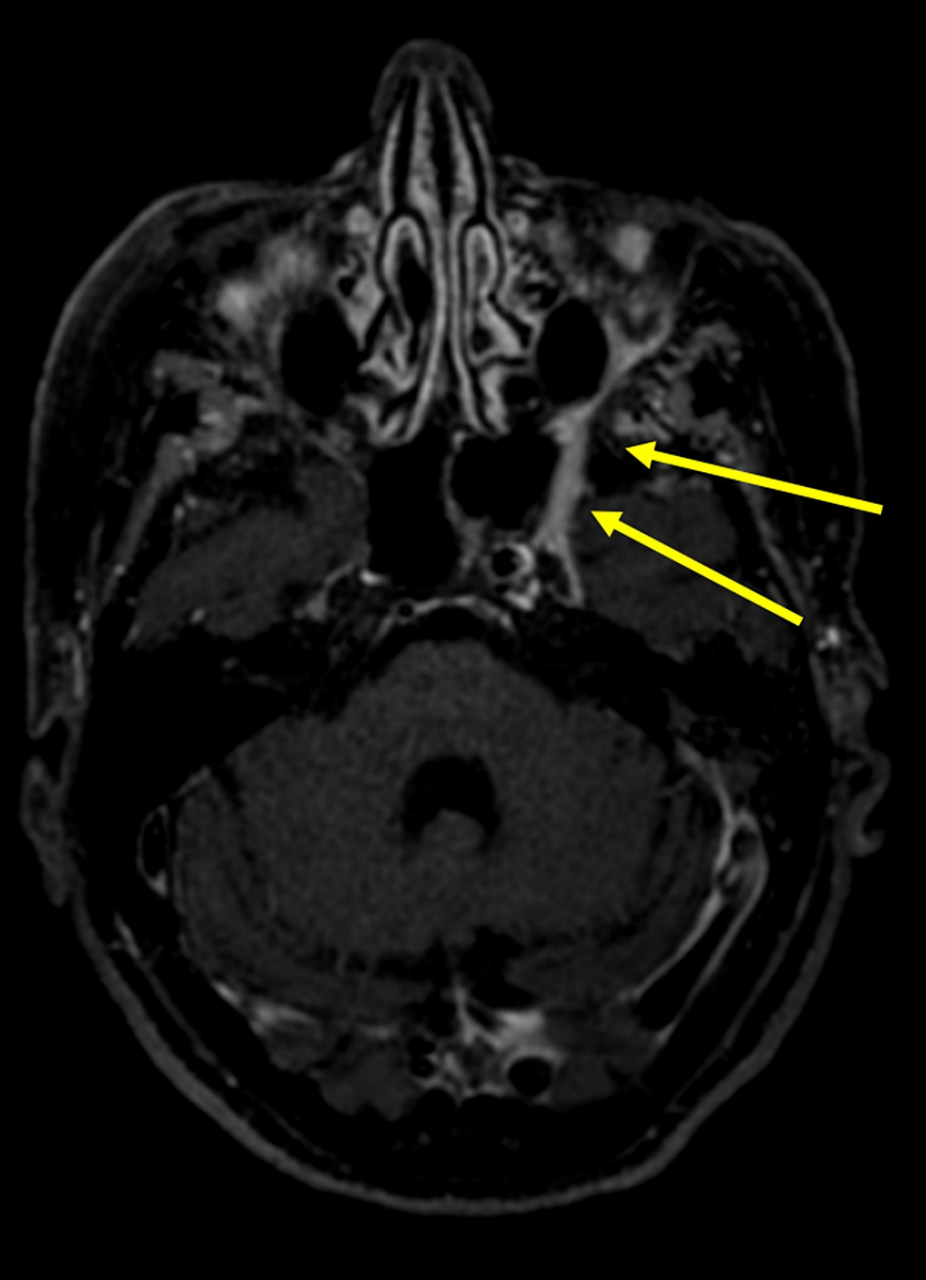
Gadolinium-enhanced axial post-contrast T1-weighted 3-Tesla MRI images of the brain showing perineural tumor spread (yellow arrows).

A second 3-Tesla high-resolution MRI of the brain and skull base with and without contrast showed far more detail than the lower 1.5-Tesla MRI with abnormal enhancement of the left nasolabial fold and perineural tumor spread along the left infraorbital nerve to the left pterygopalatine fossa (Figure 2).

A lumbar puncture was performed; cerebrospinal fluid (CSF) was negative for malignant cells by flow cytometry, and the CSF findings are summarized in Table 1.

**Table 1:** Results of CSF analysis. mg/dL: mg per deciliter, IgG: immunoglobulin G

Test	Result	Reference range	Interpretation
Protein (mg/dL)	26.9	0-44	Normal
Glucose (mg/dL)	59	40-70	Normal
IgG, quantitative (mg/dL)	1.9	0-6.7	Normal
Albumin, CSF (mg/dL)	18	10-46	Normal
Immunoglobulin G, serum (mg/dL)	801	586-1602	Normal
Albumin	3.9	3.7-4.7	Normal
IgG/albumin ratio	0.11	0-0.25	Normal
IgG index	0.5	0-0.7	Normal
Oligoclonal bands	1 band	<4 bands	Normal

Isoelectric focusing (IEF) and immunoblotting were used to perform oligoclonal banding testing. One paired band and zero nucleated cells were found in both the CSF and serum, which indicates an inflammatory process outside the central nervous system. Zero oligoclonal bands were found in the CSF, which rules out an ongoing central nervous system inflammatory process.

A paraneoplastic panel including anti-acetylcholine receptor ganglionic neuronal antibodies, anti-amphiphysin antibodies, anti-glial nuclear antibody type 1, anti-neuronal nuclear antibody type 2, anti-neuronal nuclear antibody type 3, collapsing response-mediator protein-5 (CRMP-5) immunoglobulin G, neuronal voltage-gated potassium channel antibodies, calcium channel antibody P/Q-type, and Purkinje cell cytoplasmic antibodies were negative. A referral to the ear-nose-throat (ENT) specialist revealed no cervical adenopathy or neck masses. A computed tomography (CT) scan of the chest, abdomen, and pelvis did not reveal evidence of malignancy. The patient refused a facial nerve biopsy of a facial nerve twig of the parotid gland and other invasive procedures including a skin biopsy over the nasolabial fold. It was determined that the likely source of the malignant perineural spread along the skull base foramina (foramen rotundum and ovale) was dormant basal cell carcinoma cells. The patient received proton beam radiation therapy to the involved sites, including the maxillary region of the face, the involved sinuses, and the skull base.

## Discussion

Neoplastic involvement of the trigeminal nerve can cause TNp and can pathologically be attributed to nerve compression, perineural spread (PNS), and/or perineural invasion (PNI). Metastasis from breast or lung cancer to the Gasserian ganglion is rare, as is leptomeningeal metastasis, which is usually lymphomatous [8,10]. Carcinomatous leptomeningitis can affect other cranial nerves and can rarely present with an isolated TN and/or TNp [11]. Most primary tumors of the trigeminal nerve are due to a schwannoma and rarely are due to meningioma, lipoma, or epidermoid tumor [12].

The peripheral and cranial nerves harbor three layers of connective tissue across their diameter: endoneurium, perineurium, and epineurium. The perineural space is located between the nerve axon and the perineural layer. This potential space can allow for tumor spread and growth. There are two types of perineural tumor growth: perineural invasion (PNI) and perineural spread (PNS). PNI is defined by malignant cells invading the perineural space and is a histological diagnosis. PNS is a radiological diagnosis of malignant spread along the nerve and is identified by enhancement detected by high-resolution MRI. During the initial stages of the disease, only PNI may be present. As disease progression occurs, PNI can become PNS, which is clinically more aggressive [13,14]. A patient with numbness to the territory innervated by the mental branch of the mandibular nerve should always raise a red flag. The mental branch is purely sensory in function and provides cutaneous innervation to the lower lip and chin. This characteristic numbness is aptly named the numb-chin syndrome. Malignant mental neuropathy can indicate the presence and/or recurrence of a malignant tumor [15]. Facial numbness that spreads and radiates into the tongue, roof of the mouth, and inside the cheeks should raise a high index of suspicion for an inflammatory or malignant invasion of the trigeminal nerve.

Hence, perineural tumor spread of carcinoma may be seen along the maxillary and mandibular division of the trigeminal nerve. Nerve enhancement is one of the radiological findings in perineural tumor spread and can serve as a dependable sign. High-resolution MRI is the imaging modality of choice due to its ability to demonstrate soft tissue in high detail. MRI enhancement of the mandibular nerve in asymptomatic patients is very uncommon and was only seen in 3% of patient cases without symptoms of TNp. Therefore, when symptomatic maxillary or mandibular nerve enhancement is discovered on MRI, a high index of suspicion for an underlying disease process should be raised [16,17].

Perineural spread of head and neck cancers is a well-described phenomenon. At the cellular level, neural secretion of glial-derived neurotrophic factor (GDNF) may allow perineural spread. GDNF phosphorylates the rearranged during transfection (RET) tyrosine kinase receptor that triggers downstream signaling pathways that allow malignant cell migration [18]. Adenoid cystic carcinoma and squamous cell carcinoma are the most common malignancies that can spread perineurally [3]. Although not the most common, basal cell carcinoma has been described in the literature. However, very few cases exist where the only clinical manifestation of basal cell carcinoma recurrence is trigeminal neuropathy [19].

Additional etiologies of TNp include inflammatory, autoimmune, paraneoplastic, and infectious disorders. Inflammatory/autoimmune etiologies include sarcoidosis, Lyme’s disease, Sjogren’s syndrome, and systemic lupus erythematosus [8]. Malignancies that can cause paraneoplastic syndrome include, but are not limited to, small cell lung cancer, breast cancer, ovarian cancer, and pancreatic cancer. TNp can be one of the first clinical manifestations of a paraneoplastic syndrome [20]. Infectious causes include leprosy, herpes simplex virus, varicella-zoster virus, actinomycosis, and aspergillus [8].

## Conclusions

In conclusion, the diagnosis of trigeminal neuropathy should evoke a specific set of differential diagnoses. Since trigeminal neuropathy can be one of the first and only manifestations of a head and neck tumor, local malignant perineural invasion should be seriously considered. A high-resolution MRI of the skull base with and without gadolinium contrast enhancement should be ordered as our case demonstrates that a higher magnetic field strength can highlight pathology not seen with lower magnetic field strength MRI.
